# Creating a Basic Ethical Framework for Digital Lifestyle Interventions: A Narrative Review

**DOI:** 10.1016/j.mcpdig.2025.100295

**Published:** 2025-10-14

**Authors:** Nicolien D.M. Dinklo, Maartje H.N. Schermer, Ineke Bolt, Hafez Ismaili M’hamdi

**Affiliations:** a Erasmus Medical Center, Department of Public Health, Rotterdam, Netherlands; b Erasmus University Rotterdam, Erasmus School of Health Policy, and Management, Rotterdam, Netherlands; c Maastricht University, Department of Health, Ethics and Society, Maastricht, Netherlands

## Abstract

Digital tools are often seen as promising avenues for promoting and sustaining healthy lifestyle behaviors. They not only offer benefits such as personalization, scalability, and cost-effectiveness but also raise significant ethical concerns. Issues such as equitable access, informed consent, and fair outcomes, particularly for vulnerable populations, must be addressed. An ethical framework is needed to guide the creation of digital lifestyle interventions. A narrative review was conducted across 3 domains: (1) general ethical principles for public health interventions, (2) ethical frameworks for lifestyle interventions, and (3) ethical considerations for digital tools in health promotion. A total of 16 articles were found across all 3 inclusion domains. The following 5 core ethical themes were identified: (1) respect for autonomy; (2) beneficence; (3) harms; (4) equity; and (5) responsibility, sustainability, and accountability. Two ethical considerations stood out in the context of digital interventions: health equity and privacy. Although digital tools may be an effective form of lifestyle intervention, they can disproportionately benefit individuals already in advantaged positions. We present a basic ethical framework for guiding the development and deployment of these digital tools. The framework highlights the tensions that may arise between competing ethical principles and helps developers determine which considerations are most relevant, and to whom, at different stages of intervention design and development.

**Article Highlights**

- This study presents an ethical study into existing ethical frameworks for public health and lifestyle interventions.
- It examines the ethical considerations for the use of digital tools in health promotion.
- It introduces a basic ethical framework for digital lifestyle interventions.

Lifestyle improvement interventions may represent a promising strategy for reducing health inequities and improving the overall health of a population.^1^^,^^2^ Health disparities are not merely unfortunate byproducts of the inequalities in opportunities to pursue a healthy and good life, but rather, they are increasingly recognized and rightfully labeled as injustices.^3^ Vulnerable populations, who often experience lower socioeconomic status, limited education, and adverse environmental conditions, face significant disparities and inequities in health.^4^^,^^5^ Although promoting a healthy lifestyle offers a practical route to better health outcomes, especially when paired with efforts to mitigate socioeconomic disadvantages, achieving and sustaining such changes are notoriously difficult.

Digital tools are an emerging means to promote and sustain healthy lifestyle behaviors at scale. For example, in the periconception period, the mHealth program Smarter Pregnancy provides users with tailored lifestyle coaching aimed at optimizing health before, during, and after pregnancy.^6^ Other mHealth interventions, such as Eat smarter with your child^7^ and Pregnancy,^8^ are designed to help young parents make informed dietary choices, either for themselves during pregnancy or for their kids. Additionally, Groeigids^9^ offers a chat function that connects parents with youth nurses who can answer questions about their child’s health. Research suggests that such programs can lead to measurable improvements in lifestyle choices, such as an increase in vegetable intake among users.^10^

Nevertheless, digital lifestyle tools also require constant attention to ethical concerns, including the need to ensure fair access and equitable outcomes, particularly for vulnerable populations,^11^ as well as securing informed consent for their use.^12^ These challenges place such interventions at an ethical crossroads, where their potential benefits must be weighed against the risks of perpetuating existing health inequities or failing to achieve meaningful results. Furthermore, the true efficacy of lifestyle interventions remains underexplored. This highlights the need of an ethical framework to guide developers, policymakers, and public health professionals to identify the conditions under which digital lifestyle interventions (DLIs) are ethically justifiable. Key ethical concerns include respecting user autonomy, ensuring interventions genuinely improve health outcomes, avoiding harm, and promoting equity. Additionally, the collection of sensitive personal data by digital tools raises ethical questions about obtaining adequate informed consent and ensuring fair, unbiased, and transparent data use.^13^

To address these challenges, it is essential to identify the converging and diverging ethical considerations surrounding DLIs. Currently, there is no common ethical framework for DLIs in public health. Therefore, this article aims to develop a basic ethical framework, providing a structured approach to navigate the unique ethical complexities of DLIs. To build this framework, we analyzed the existing literature to identify the key ethical considerations relevant to the development and use of DLIs designed to improve public health. This framework provides guidance for developers, policymakers, and public health professionals on how to develop and deploy digital tools in a way that promotes both individual well-being and social justice. Ultimately, we propose a basic framework that presents ethical considerations related to DLIs that developers, policymakers, and public health professionals could use to ensure that their digital lifestyle tools are ethically sound.

## Methods

### Search Strategy

To provide a comprehensive overview of ethical considerations relevant to DLIs, we conducted a narrative review with 3 separate literature searches starting in June 2023 with additions and updates in August 2024.^14^ Searches were conducted in 4 databases commonly used in bioethics research: Medline, Embase, Web of science core collection, and Google Scholar. An information specialist supported the development of search terms and strategies.

The searches were organized around 3 main areas of ethical relevance:

1. General ethical principles in public health interventions, using the keywords “life style AND public health” AND “ethics” (Supplemental Appendix 1, available online at https://www.mcpdigitalhealth.org/).
2. Ethical frameworks for lifestyle interventions, using the keywords “lifestyle” AND “ethical framework” (Supplemental Appendix 2, available online at https://www.mcpdigitalhealth.org/).
3. Ethical considerations for digital tools in health promotion, using the terms “ethics” AND “digital” AND “health promotion” (Supplemental Appendix 3, available online at https://www.mcpdigitalhealth.org/).

### Article Selection

Included articles discussed ethical considerations, such as principles, values, frameworks, or normative analyses or evaluations, which could be used to critically assess the use of public health/lifestyle interventions or digital tools for health promotion. We focused on articles addressing preventive rather than clinical contexts. Articles were excluded if they were not available in full text, not in English or Dutch, or limited to conference abstracts. Articles that focused specifically on artificial intelligence (AI) without a direct link with a DLI were excluded due to the distinct and expansive ethical challenges associated with that topic, which fall outside the scope of this review.

Given the breadth of the topic and variability in how ethical considerations or frameworks are addressed across the literature, article selection combined formal search results with knowledge of the field by the authors. Titles and abstracts were scanned for relevance, and additional key articles were identified through gray literature by citation tracking and familiarity with the literature. Because this is a narrative review, the goal was not to exhaustively identify every eligible publication, but to synthesize key ethical considerations from a conceptually rich and relevant body of literature. The selection provides a robust and representative foundation for thematic analysis.

Initial screening and selection were performed by one author (N.D.M.D.), with inclusion decisions discussed and validated with another (H.I.M.). Disagreements were resolved through consensus. Sixteen articles were included (see Supplemental Table 1 for included articles, available online at https://www.mcpdigitalhealth.org/):

• Eight articles on general ethical principles for public health interventions;
• Two articles on ethical frameworks for lifestyle interventions;
• Six articles on ethical considerations for digital tools in health promotion.

### Data Analysis

A thematic analysis^15^ was conducted by author (N.D.M.D.), who coded the ethical content of the included articles. These codes were organized into overarching themes and subthemes, which were refined through discussions with other authors (H.I.M., M.H.N.S., and I.B.). All themes and their related articles are shown in Table.^16^, ^17^, ^18^, ^19^, ^20^, ^21^, ^22^, ^23^, ^24^, ^25^, ^26^, ^27^, ^28^, ^29^, ^30^ These findings were further synthesized into a basic framework of ethical criteria for DLIs. The validity of our framework is warranted because it is essentially based on generally accepted and widely used ethical frameworks.

**Table:** Overview of Subthemes

Theme	Subtheme	Description	Reference
Respect for autonomy	Informed consent	Honoring and supporting each person’s right to make informed, voluntary decisions free from coercion or undue influence	16, 17, 18, 19, 20, 21, 22, 23, 24, 25
	Empowerment	Providing individuals with the knowledge, skills, and resources needed to actively participate in and take control of their health care decisions	16,17,19, 20, 21,25, 26, 27, 28
	Privacy	Protecting participants information and data	16, 17, 18, 19, 20, 21, 22, 23, 24, 25
	Transparency	Communicating the intervention’s purpose and data usage	20,24,25,29
	Building and maintaining trust	Fostering cooperation through honest communication and striving to be worthy of trust	13,17,20,24,25
Beneficence	Effectiveness	Ensuring the intervention achieves its intended goals	16,17,19,25,29,30
	Benefits and burdens ratio	Ensuring that the intervention is capable of producing benefits (doing good) and these benefits should outweigh any burdens it creates	16,18,19,27,24,25,29,30
Harms and accountability	Preventing harm	Avoiding or reducing risks and adverse consequences of the intervention	16,17,19,24,25
	Stigmatization and discrimination	Avoiding harmful stereotypes or exclusion of groups	16, 17, 18, 19,21,26
Equity	Fairness: benefits and burdens	Ensuring equitable distribution of benefits and burdens	16,17,19,24,30
	Cost-effectiveness	Achieving meaningful impact relative to costs	16,19,26,29,30
	Health equity	Reducing or avoiding disproportionate disadvantages among groups	16, 17, 18, 19, 20,24,25,26, 27, 28, 29
	Algorithmic bias	Preventing exclusion or unfair treatment owing to biased algorithms	20
Responsibility, sustainability, and accountability	Responsibility	Assigning responsibility for health	17,18,25,26
	Environmental sustainability	Acting responsibly to preserve resources for future generations and preserve the health of the planet	25,26
	Sustainability	Ensuring long-term viability of the intervention	17,26,27
	Accountability	Ensuring the individuals in positions of authority to justify their actions and decisions, particularly regarding how they fulfill their responsibilities within this role	24, 25, 26,29

## Results

We found 5 main ethical themes, each with its own subthemes. The 5 main themes are as follows: (1) respect for autonomy, (2) beneficence, (3) harms, (4) equity, and (5) responsibilities, sustainability, and accountability. The Table provides a short description of the subthemes, along with a list of the literature each subtheme was identified in and in which area of inclusion it was observed.

### Theme 1: Respect for Autonomy

#### Subtheme 1: Informed Consent

Respecting individual autonomy requires obtaining informed consent before participants partake in a program aiming to influence lifestyle behaviors, making sure that a voluntary decision is made, free from coercion, manipulation, or undue influence.^16^^,^^17^ Informed consent involves not only providing participants with information about the intervention but also ensuring they fully understand what they are agreeing to.^18^^,^^19^

Within the digital environment, informed consent is especially relevant when it comes to data sharing. Digital tools require additional caution to address concerns about data security and potential technology misuse.^20^ Several articles highlight the concern of a possible lack of participant awareness of data collection and storage compromising their informed consent.^21^^,^^22^ When participants agree to an intervention, they may understand that they are sharing data but not the extent of data that are collected or where and how long they are being stored. Segura Anaya et al^22^ described how data mining is used for marketing purposes, thereby misusing personal information. Knowledge of this misuse can help address privacy issues and make users aware before providing informed consent.

#### Subtheme 2: Empowerment

Tannahill^26^ defines empowerment as, “Empowerment is about enabling people to have a greater degree of self-determination in relation to their health and involves restrictions to the freedom of individuals and corporate entities to expose others to risk of harm.” Empowerment is a form of self-governance, which interventions can support by helping participants achieve their goals that aligns with their own values.^16^^,^^27^ For example, in case people want to stop smoking, a smoking cessation intervention should promote intrinsic motivation, to help participants realize the goals they have set for themselves. Targeting intrinsic motivation will additionally help sustain the cessation without just relying on extrinsic motivations such as rewards.^19^

Nickel et al^28^ remark that digital health interventions “often fail to be empowering in any plausible sense” because they can cause a loss of power in participants. Participants may not be aware of which data are being collected, where they are stored, and how they are used. This removes or hinders the participant’s control over their data. In order for participants to be capable of benefiting from the intervention, they must be motivated and empowered not only through information but also through practical means (eg, by teaching digital skills).^21^

#### Subtheme 3: Privacy

Privacy is critical in interventions to ensure participants’ information remains confidential. Participants must have control over who accesses their data, so providing informed consent to allow their data to be accessed by particular parties is essential.^16^^,^^18^ For example, smoking cessation incentives should avoid personal details that link users to smoking behavior.^19^ Any collected data must be safeguarded against third-party access, and secondary use is only permissible with explicit consent.^20^^,^^3^ The rise of digital interventions has amplified privacy concerns owing to the large-scale collection of sensitive data, such as passive data tracking.^24^

#### Subtheme 4: Transparency

Potential participants in a lifestyle intervention should be given information about the purpose of the intervention and the way their data are handled. A transparent intervention is one where the participants are kept informed of decisions that have been or are being made about the goal of the intervention and the method to achieve this. A digital intervention should be transparent about the data it collects as well as the way they are used. This can raise awareness and enhance the participant’s trust in the intervention.^31^

#### Subtheme 5: Building and Maintaining Trust

Trust is a crucial ethical concern in public health interventions because it directly affects both the success and the integrity of the intervention. When individuals trust the goals and intentions behind an intervention, they are more likely to engage with it voluntarily and give informed consent. Designers and developers of an intervention must therefore strive to be trustworthy.^24^ Interventions that lack trust risk causing harm, whether through poor communication, breach of privacy, or perceived negative outcomes. This can lead to skepticism or refusal to participate, undermining the intervention’s goals and potentially harming future public health efforts. The digital environment allows for easy dissemination of misinformation or false information that affects how the public views digital health information or promotion. Generating and maintaining trust is therefore more difficult and essential for the public to benefit from digital interventions.^13^^,^^20^

### Theme 2: Beneficence

#### Subtheme 1: Effectiveness

Before implementation, an assessment of effectiveness must be conducted to ensure that the intervention is genuinely capable of achieving its intended purpose and is suitable for helping the participant.^19^^,^^25^ This evaluation is crucial in establishing the ethical justification for the intervention because it confirms that the intervention can safeguard and promote health as intended. Without demonstrated effectiveness, the intervention lacks the ethical foundation required to proceed because it may fail to meet the basic standards of beneficence.^30^

#### Subtheme 2: Benefit and Burden Ratio (Proportionality)

An intervention must be capable of producing benefits (doing good), and these benefits should outweigh any burdens it creates.^16^^,^^24^^,^^26^ For instance, a program that aims to reduce overweight or obesity might inadvertently create stigma. These inadvertent consequences do not make the intervention a priori unjustifiable, rather, following the demand of proportionality, these psychological burdens should be weighed against the physical benefits pertaining to weight loss.^18^^,^^32^

### Theme 3: Harms

#### Subtheme 1: Preventing Harm

A beneficial intervention may still pose certain health risks or burdens, including adverse consequences that only emerge after implementation.^16^ This possibility necessitates proactive measures to prevent or minimize harm wherever possible. A harm prevention approach can be supported by taking specific precautions, such as implementing actions that are potentially reversible, thus allowing flexibility if unintended consequences arise.^25^ Furthermore, interventions should be carefully designed to avoid promoting behaviors that could conflict with their intended health goals. For instance, a smoking cessation program should avoid offering incentives so high that they might tempt nonsmokers to start smoking just to qualify for the program’s benefits.^19^

#### Subtheme 2: Stigmatization and Discrimination

Stigmatization and discrimination within public health interventions can occur when a specific health condition or behavior becomes associated with a particular group. This association can lead to negative perceptions and reinforce harmful stereotypes, particularly if the intervention fails to address the unique concerns of these groups or remains inaccessible to them.^16^ When it comes to digital tools, these risks are heightened due to the extensive data collected and shared through digital interventions. Data should be stored in such a way as to prevent unintended access, so that confidential information does not fall into the wrong hands and enable stigmatization and discrimination. Confidential information, if misused for commercial gain or shared without adequate safeguards, can further marginalize vulnerable groups and individuals, potentially prioritizing profit over the well-being of participants.^21^

### Theme 4: Equity

#### Subtheme 1—Fairness: Benefits and Burdens

An intervention must not only demonstrate a balance where benefits outweigh harms but also ensure the fair distribution of these burdens and benefits among its target audience.^16^ This evaluation aligns with the principle of distributive justice, which safeguards against the risk that certain groups may be disproportionately burdened by the intervention while receiving fewer or inadequate benefits.^24^^,^^25^ Breunis et al^19^ explain how smoking during pregnancy is highly associated with socioeconomic disadvantage. They argue that the intervention should be tailored to this group so that the intervention improves the health of those whose health is most impaired. Moreover, using incentives for smoking cessation is also beneficial to women of low socioeconomic status because the intervention delivers money instead of costing money.

#### Subtheme 2: Cost-effectiveness

Implementing an intervention that effectively induces change must also demonstrate cost-effectiveness, meaning that the intervention should provide a meaningful impact relative to its costs.^16^^,^^26^ Efficiency in this context not only involves the upfront costs of implementation but also considers the ongoing maintenance burden that the intervention might impose over time.^29^ Lifestyle interventions are primarily designed to improve the health of their users, yet they may also contribute to the broader goal of reducing overall health care costs.^19^

#### Subtheme 3: Health Equity

One of the primary goals, and indeed, ethical responsibilities, of public health is to actively work toward minimizing health inequities, which are widely considered preventable, unnecessary, and unjust.^24^^,^^27^ To maximize impact, interventions could prioritize groups that stand to benefit the most from lifestyle improvements, helping to make healthy living more accessible and equitable across diverse population segments. At the same time, focusing solely on maximizing impact may inadvertently overlook or provide less benefit to those who are worst off because these groups might face additional barriers that limit their ability to engage with or benefit fully from the intervention.^18^^,^^19^ For example, digital tools can exacerbate inequalities among disadvantaged populations who lack access, skills, or competency. The designers and developers have a shared responsibility to ensure equitable access by actively seeking to enhance the usability of digital tools for such populations.^32^

Digital intervention may inadvertently exacerbate health disparities, for example, because of differences in digital literacy and unequal access to necessary devices and software. Nickel et al^28^ notices that an indirect strategy whereby digital interventions are initially targeted at high-resource individuals because these groups may benefit more readily, potentially freeing up resources that can then be redirected to support more vulnerable populations. However, Nickel et al^28^ critiques that such an approach carries the risk of encouraging overuse of health care resources by high-resource individuals, which could ultimately divert attention and resources away from higher-need, disadvantaged groups, further entrenching health inequities.

#### Subtheme 4: Algorithmic Bias

The incorporation of computer algorithms in public health interventions, while promising for scalability and personalization, introduces the risk of algorithmic bias, which can inadvertently exclude or unfairly target certain populations, even if the intervention is designed for broad, equitable application.^20^ Algorithmic bias can originate from the design of new algorithms or be perpetuated by pre-existing societal biases embedded within the data on which these systems are trained.

### Theme 5: Responsibility, Sustainability, and Accountability

#### Subtheme 1: Responsibility

Responsibility refers to the designation of who is obligated to act and what specific actions are required.^26^ In public health, this applies to both individuals and organizations. Although individuals bear responsibility for their own health, health promotion organizations, such as health care services, public health agencies, and policymakers, have a duty to provide both curative and preventive measures, as well as to create environments that enable healthy choices. An intervention aimed at an individual should not limit the societal collective responsibility for health promotion. Have et al^18^ explains this as a “just division of responsibilities,” which not a single party is held responsible when there is a “complex web of causal factors.” Therefore, they underline that programs should “urge various actors to make efforts to make healthy choices possible, which is the only way to tackle the worldwide increase of overweight and obesity.”

#### Subtheme 2: Environmental Sustainability

Respect for the natural environment entails acting in such a way that the action can be maintained in the future and for future generations. This consideration is rarely the primary motivation for a public health intervention; However, Filiatrault et al^25^ describe it as a form of ensuring equity between generations. An intervention that is sustainable for environment should safeguard and conserve the physical environment and resources.^26^

#### Subtheme 3: Sustainability

The sustainability of an intervention relates to the long-term effect that can be achieved and whether the intervention is able to be implemented for the expected or necessary duration.^26^ Sustainability can include making appropriate adaptations to the environmental conditions (eg, provision of healthy foods) so that a changed behavior does not return to the situation it was in before.^27^

#### Subtheme 4: Accountability

Accountability pertains to the duty of individuals in positions of authority to justify their actions and decisions, particularly regarding how they fulfill their responsibilities.^25^ In the context of interventions that influence lifestyle, accountability is essential to ethically manage potential intrusions on privacy, restrictions on personal freedom, and behavioral influences aimed at improving health outcomes. Trade-offs that are made should be done openly so that proper justification can be given for the intervention and its effect. In the realm of digital tools, where data can be continuously collected and used over extended periods, accountability takes on heightened importance. Ensuring responsible data use over the long-term requires open and stringent accountability measures.^13^

## Discussion

The purpose of this research was to triangulate the ethical concerns from 3 different search areas to identify the relevant ethical concerns for an ethical framework for DLIs. In this discussion, we will further specify how the themes and subthemes we have identified in our results are relevant for DLIs.

The significance of our findings for DLIs lies in the overlap between ethical frameworks for lifestyle interventions and the added complexities of a digital environment. The issue of empowerment serves as an example. In public health, empowerment means enabling individuals to make informed choices that align with their personal goals. Digital tools are often presented as a means of empowerment by allowing individuals greater control over their health.^33^ However, this assumption fails when participants lack the resources or digital literacy required to engage with these tools.^28^^,^^34^ Socioeconomic determinants further contribute to low adoption rates, exacerbating health inequalities.^35^^,^^36^ This, in turn, risks stigmatizing those unable to engage with digital interventions, potentially labeling them as irresponsible and making the intervention both unrealistic and unfair.^37^ These issues highlight the challenges of digital tools as a mechanism for empowerment in health interventions.^38^

The example of empowerment illustrates how digital interventions introduce new ethical risks. Among these, 2 ethical considerations stand out owing to their prominence in the reviewed articles: justice (specifically, health equity) and privacy.

Justice and health equity are key concerns when transitioning interventions from physical to digital formats. Although digital tools can be beneficial because they may be cost-effective^39^ and can be more personalized,^40^ these benefits may primarily serve those already in an advantaged position. Barriers such as low health and digital literacy,^41^ the intervention being unsuited to their needs,^42^ and having no face-to-face interaction^43^ may prevent already disadvantaged groups from benefiting. A DLI designed to reduce health inequalities must therefore address these barriers and limitations. Mitigation strategies could include multilingual interfaces, compatibility with older devices, and inclusive design practices, such as usability testing with underserved populations to identify and remove accessibility barriers.^44^

A key risk related to health equity in digital tools is algorithmic bias. Unlike traditional interventions, digital tools rely on algorithms, including those that do not use AI (under some definitions), which can unintentionally reinforce disparities.^27^ Addressing these biases requires targeted ethical scrutiny beyond what standard public health frameworks provide.

Privacy, particularly concerning personal health information, is another ethical priority that is heightened in a digital context. Digital tools frequently collect, analyze, and store sensitive health data,^22^ making it insufficient to simply claim that a tool respects privacy. Ethical considerations should specify how data are handled, stored, and shared, as well as the purpose and duration of retention. For instance, in a DLI, privacy could be safeguarded through limited data storage durations, robust informed consent processes, and user-controlled data access settings. Establishing clear, user-friendly privacy settings that allow users fine-grained control over their data sharing would further ensure meaningful privacy protection.^22^

There are also a few ethical considerations that were sparsely found in any of the included articles, namely that of sustainability, and respect for the environment. Although not explicitly addressed in ethical frameworks for lifestyle interventions or the literature we found on the ethical considerations for the use of digital tools for such interventions, their relevance cannot be dismissed. Moreover, although sustainability may seem peripheral to digital lifestyle tools, it speaks to the broader ethical obligation to consider the long-term impact of health interventions, particularly in terms of environmental and resource implications. A digital tool designed for lifestyle improvement should ideally be sustainable, both in its technical infrastructure and in its alignment with planetary health.^11^^,^^45^

### Creating an Ethical Framework

The aforementioned ethical considerations require further specification when applied to DLIs, since existing frameworks are too general to capture the distinctive challenges posed by these technologies for lifestyle interventions. Our framework is derived systematically from established ethical frameworks in public health and the literature on digital tools in health promotion, with attention to core ethical values such as autonomy, justice, beneficence, nonmaleficence, and accountability. We have added concrete questions within these themes tailored to DLIs such as: questions related to environmental sustainability, equity, bias, digital literacy, and the attention for individual vs collective responsibility. In this way, our framework differs from already existing approaches by both retaining core ethical commitments and explicitly specifying them by means of concrete guiding questions, to the realities of DLIs. Our framework offers users guidance on how to anticipate and mitigate ethical risks during development and deployment of DLIs, enabling them to translate abstract principles into actionable practices.

### Ethical Framework for DLIs

#### Respect for Autonomy

• How is informed consent for the DLI, the data collection, and data use considered?
• How does the DLI affect voluntary action and decisions by the user?
• How is the participant empowered to make choices that align with personal values by using the DLI?
• How is the privacy of the participant considered, communicated, and respected?
  o Is the participant able to choose what information is shared?
• How is the purpose of the DLI, to improve their lifestyle, and the use of the data made transparent?
• How does the DLI make sure it warrants the trust of the users?

#### Well-being

• Is the DLI effective in improving lifestyle behaviors?
  o How does the DLI promote a sustainable lifestyle?
• How do the potential harms outweigh the expected benefits, to the individual and society?
• How does the DLI avoid or minimize potential negative consequences, also to those not directly involved in the DLI?
• How does the DLI avoid causing undue stigmatization or discrimination?

#### Equity

• How does the DLI distribute the benefits and burdens fairly?
• Is the DLI cost-effective?
  o Is the DLI more cost-effective in comparison with a physical intervention?
• How does the DLI affect health inequity and act to reduce this where appropriate?
  o How are barriers to the use of DLIs considered?
• How is the DLI designed to be accessible, also to those with limited access to technology or limited digital literacy?
• How is the DLI designed and executed in a way that the target group can benefit from it?
• How are the algorithmic biases considered?

#### Responsibility, Sustainability, and Accountability

• How does the DLI ensure that the individual nature of the intervention does not disregard the collective responsibility to safeguard and promote health?
• How does the DLI take into account the environmental impact of a digital intervention?
• How does the DLI consider accountability?
  o Are the trade-offs that are made done openly, and are reasons for these trade-offs made transparent to those affected by the decisions so that proper justification is considered for the intervention and its effects?

### How to Use the Framework

**Figure 1:**
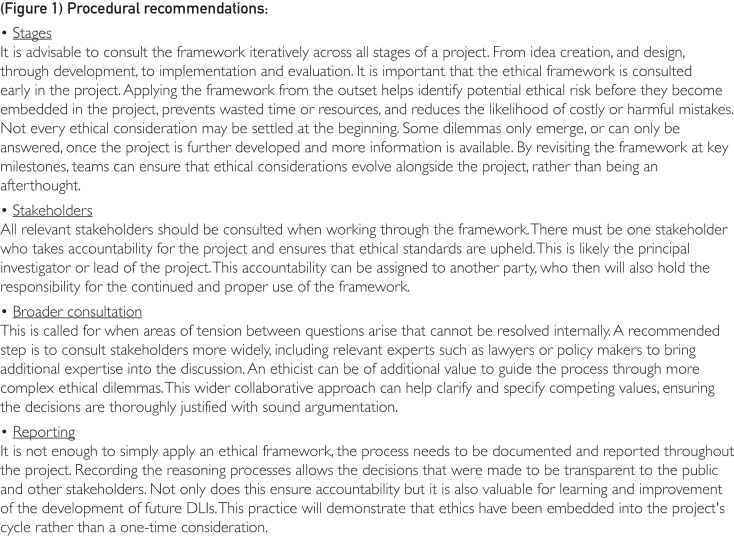
Procedural recommendations: a guide for users of the ethical framework.

It is important to recognize that a key limitation of many ethical frameworks, including ours, is that they can only aid in the identification of (potential) ethical issues and tensions so they may be properly addressed when developing and deploying an intervention—the framework itself does not solve these issues or tensions. Some frameworks, such as ours, may appear to function as a checklist, allowing ethical criteria to be ticked off along the way until a final ethically justifiable intervention is created. However, if ethical decision making were truly this straightforward, the need for such frameworks would be far less pressing. An ethical framework does not provide a single right answer and must therefore be used critically. The framework is designed to be applied across all stages of the development and deployment process of the intervention, rather than at a single point in time. Although the framework is designed to be applied directly without requiring the involvement of an ethicist, ethicist may also act as a stakeholder. Their input can be valuable when resolving complex ethical dilemmas. All questions within the framework must be considered and developers should be able to provide a response, whether through a well-reasoned justification, an explanation of how the issue has been addressed, or a description of adaptations that will be made to the product. To further guide the use of the framework, we have added procedural recommendations found in Figure 1. Our framework serves as an overview of the relevant ethical considerations for DLIs, but the real challenge lies in the practical application of the ethical requirements (norms) it entails.

**Figure 2:**
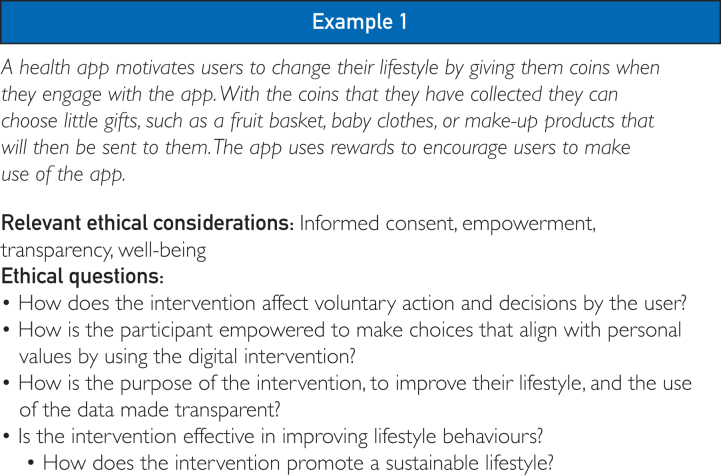
Example 1: an example of a scenario where the ethical framework can be applied.

At each stage of planning, the framework should be consulted to identify potential tensions between ethical requirements. Although some issues may be relatively simple to resolve, others may lead to complex ethical dilemmas. Resolving tensions may require making trade-offs. An intervention that maximizes overall well-being may not always be equitable, whereas one that prioritizes equity may not improve everyone’s well-being equally. To support the use of the framework, we have provided several scenarios as examples of situations where tensions might arise between the ethical considerations. For instance, in Figure 2, example 1, the intervention aims to promote a healthier lifestyle while ensuring the participants are not unduly influenced, limiting their freedom of choice. This highlights the challenge of balancing public/individual health benefits with respect for autonomy in policy design.

**Figure 3:**
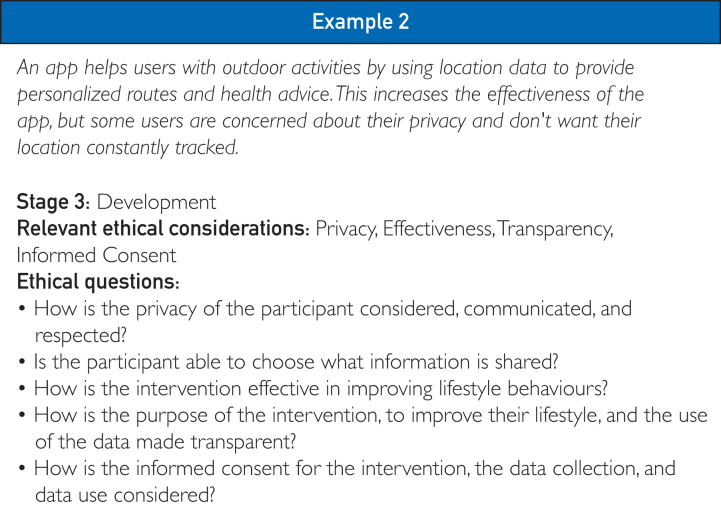
Example 2: an example of a scenario where the ethical framework can be used at different stages of the design and deployment.

There are several stages involved in the planning of DLIs, which can be summarized as follows: (1) idea creation, (2) preparation, (3) development, (4) testing, (5) implementation, and (6) evaluation.^46^, ^47^, ^48^, ^49^, ^50^ At each stage, the framework should be consulted to identify potential ethical tensions and determine whether any action is needed to address them. For example, in Figure 3, example 2, during the development stage, optimizing the use of an mHealth intervention requires the application to track user data. This creates a conflict between privacy and effectiveness, raising concerns about transparency and informed consent. Ensuring that users provide proper informed consent and that the application is transparent about data usage can help mitigate privacy risks while maintaining the intervention’s effectiveness.

**Figure 4:**
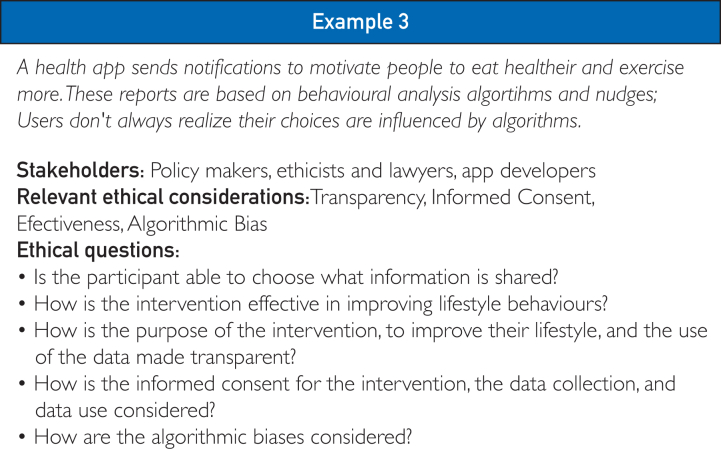
Example 3: an example of a scenario where the ethical framework can be used and the relevant stakeholders involved.

At each stage of DLI planning, different stakeholders play a crucial role. The involvement of stakeholders with specialized knowledge and resources is essential for creating a successful and well-integrated intervention. For example, application developers are most relevant during the development and testing stages, while community leaders may have a greater role in the design and implementation stages. In Figure 4, example 3, determining how to influence users without being overly manipulative requires collaboration among various stakeholders. Ethicists can assess which nudges may be ethically problematic, lawyers can ensure compliance with legal regulations, policymakers can provide guidance on existing policies and potential adaptations, and application developers can contribute practical insights into the implementation of nudges. This list is not exhaustive because different expertise may be required depending on the intervention. Moreover, while some stakeholders may be more directly involved at specific stages, this does not mean they are irrelevant throughout the entire process or that they are exempt from accountability. The designation of stakeholders to particular stages simply reflects their specialized knowledge, expertise, or resources, which allows them to better assess and balance the ethical considerations outlined in the framework at those stages.

Examining these examples and recognizing that conflicts arise between the considerations in the framework is the first step toward successfully using it. The framework serves as a valuable reminder to stakeholders, helping them identify when specific ethical considerations are most relevant and to whom they apply, providing guiding questions to draw attention to their significance. Since conflicting principles and values must be acknowledged and addressed, it is essential to designate a group that can be held accountable for the outcome of the intervention.

### Limitations

This study had several limitations. First, although we used multiple search strategies to capture a broad scope of relevant literature, this approach may have inadvertently led to the omission of some studies. To maintain focus, we not only excluded literature with a primary emphasis on AI that did not directly address DLIs but also included a broad range of articles on digital interventions, even when not explicitly framed as lifestyle related. Second, for the sake of clarity and coherence, we organized the findings into themes and subthemes. As a result, some ethical considerations, which could reasonably fit under multiple thematic categories, had to be placed under one. The decision of organization will not compromise the relevance or applicability of the final framework presented.

## Conclusion

This review has outlined the key ethical considerations for DLIs, drawing from articles that discuss general ethical principles for public health interventions, ethical frameworks for lifestyle interventions, and ethical considerations for the use of digital tools in health promotion. Although broad ethical principles such as respect for autonomy, health equity, and preventing harm remain central, the digital context introduces additional challenges, including health equity, privacy, and data use. Our findings highlight the need for an ethical framework that not only addresses these considerations but also presents a guide on how to use it.

Rather than serving as a simple checklist, the framework should be integrated throughout the intervention’s development and deployment, recognizing that ethical dilemmas will inevitably arise. Ensuring fair and just implementation requires continuous reflection and collaboration among stakeholders. By embedding ethical considerations at each stage, DLIs may be designed to be both effective and ethically sound.

## Potential Competing Interests

The authors report no competing interests.
