# Supplementary material for: Creating a Basic Ethical Framework for Digital Lifestyle Interventions: A Narrative Review
Source: Mayo Clin Proc Digit Health. 2025 Oct 14;3(4):100295. doi: 10.1016/j.mcpdig.2025.100295 (PMC12648102; doi:10.1016/j.mcpdig.2025.100295)
Supplement: Supplemental Appendix 1 [file mmc1.pdf]

Supplemental Appendix 1

General ethical principles in public health interventions

Database searched	Platform	Years of coverage	Records	Records after duplicates removed
Medline ALL	Ovid	1946 - Present	240	239
Embase	Embase.com	1971 - Present	363	268
Web of Science Core Collection*	Web of Knowledge	1975 - Present	96	30
**Total**			**699**	**537**

*Science Citation Index Expanded (1975-present) ; Social Sciences Citation Index (1975-present) ; Arts & Humanities Citation Index (1975-present) ; Conference Proceedings Citation Index- Science (1990-present) ; Conference Proceedings Citation Index- Social Science & Humanities (1990-present) ; Emerging Sources Citation Index (2005-present)

No other database limits were used than those specified in the search strategies

Excluded publication types were conference abstracts

The search was limited to the English and Dutch language

Which ethical frameworks are available for public health interventions?

zoekelementen:

1. life style intervention/program (life style AND public health)
2. ethics

Embase 363

('lifestyle modification'/de OR 'lifestyle intervention'/de OR 'healthy lifestyle'/de OR ('public health'/de AND lifestyle/de) OR (((lifestyle* OR life-style*) NEAR/3 (intervention* OR healthy OR factor* OR modif* OR change*))):ab,ti,kw) **AND** (ethics/de OR 'medical ethics'/de OR 'virtue ethics'/de OR 'bioethics'/de OR 'ethical decision making'/de OR (((code* OR framework* OR evaluation* OR guideline* OR principle* OR directiv* OR decision*) NEAR/3 (ethic* OR moral OR normative))):ab,ti,kw OR ethic*:ti) NOT ([Conference Abstract]/lim OR [Conference Review]/lim) AND ([ENGLISH]/lim OR [DUTCH]/lim)

Medline 240

(exp Healthy Lifestyle/ OR (exp Public Health/ AND exp Life Style/) OR (((lifestyle* OR life-style*) ADJ3 (intervention* OR healthy OR factor* OR modif* OR change*))).ab,ti,kf.) **AND** (Ethics/ OR exp Ethics, Medical/ OR exp Bioethics/ OR exp Ethical Review/ OR exp Ethical Analysis/ OR (((code* OR framework* OR evaluation* OR guideline* OR principle* OR directiv* OR decision*) ADJ3 (ethic* OR moral OR normative))).ab,ti,kf. OR ethic*.ti.) NOT (news OR congres* OR abstract* OR book* OR chapter* OR dissertation abstract*).pt. AND (english.la. OR dutch.la.)

Web of Science 96

TS=(((lifestyle* OR life-style*) NEAR/2 (intervention* OR healthy OR factor* OR modif* OR change*))) **AND** (TS=(((code* OR framework* OR evaluation* OR guideline* OR principle* OR directiv* OR decision*) NEAR/2 (ethic* OR moral OR normative))) OR TI=ethic*) NOT DT=(Meeting Abstract OR Meeting Summary) AND LA=(English OR Dutch)
