# Supplementary material for: Creating a Basic Ethical Framework for Digital Lifestyle Interventions: A Narrative Review
Source: Mayo Clin Proc Digit Health. 2025 Oct 14;3(4):100295. doi: 10.1016/j.mcpdig.2025.100295 (PMC12648102; doi:10.1016/j.mcpdig.2025.100295)
Supplement: Supplemental Appendix 2 [file mmc2.pdf]

Supplemental Appendix 2

Ethical frameworks for lifestyle interventions

Database searched	Platform	Years of coverage	Records	Records after duplicates removed
Medline ALL	Ovid	1946 - Present	657	653
Embase	Embase.com	1971 - Present	410	135
Web of Science Core Collection*	Web of Knowledge	1975 - Present	349	170
**Total**			**1416**	**958**

*Science Citation Index Expanded (1975-present) ; Social Sciences Citation Index (1975-present) ; Arts & Humanities Citation Index (1975-present) ; Conference Proceedings Citation Index- Science (1990-present) ; Conference Proceedings Citation Index- Social Science & Humanities (1990-present) ; Emerging Sources Citation Index (2005-present)

No other database limits were used than those specified in the search strategies

Excluded publication types were conference abstracts

The search was limited to the English and Dutch language

Which ethical frameworks are available for public health interventions that target lifestyle?

Zoekelementen:

1. Lifestyle
2. Ethical framework

Medline

(exp * Healthy Lifestyle/ OR * Life Style/ OR (lifestyle* OR life-style*).ti.) AND (* Ethics/ OR exp * Ethics, Medical/ OR exp * Bioethics/ OR exp * Ethical Review/ OR exp * Ethical Analysis/ OR ethic*.ti.) AND (english.la. OR dutch.la.)

(exp Healthy Lifestyle/ OR exp Life Style/ OR exp "Tobacco Use"/ OR exp Smoking Cessation/ OR Health Behavior/ OR exp Tobacco Products/ OR exp Diet/ OR exp Drinking/ OR exp Drinking Behavior/ OR exp Eating/ OR Feeding Behavior/ OR Malnutrition/ OR exp Micronutrients/ OR

exp Obesity/ OR Carbohydrates/ OR "Diet, Food, and Nutrition"/ OR Nutritional Sciences/ OR Nutritional Status/ OR Exercise/ OR Nutrients/ OR Sedentary Behavior / OR Sitting Position/ OR Sports/ OR "Physical Education and Training"/ OR exp Sleep/ OR exp Sleep Wake Disorders/ OR exp * Public Health/ OR * Health Promotion/ OR (lifestyle* OR life-style* OR smoking OR tobacco* OR cigarette* OR diet* OR eating OR drinking OR (food* ADJ3 intake*) OR malnutrit* OR macronutrient* OR trace-element* OR micronutrient* OR Meal-patter* OR ((calor* OR sugar* OR fat OR Protein* OR alcohol*) ADJ3 (intake* OR consumption*)) OR obes* OR overweight* OR body-weight OR body-mass OR bmi OR carbohydrate* OR Vegetarian* OR vegan* OR Low-carb* OR nutrition* OR Binge-drink* OR (Alcohol* ADJ3 abuse) OR alcoholism* OR (physical* ADJ3 (activ* OR inactiv* OR exercise OR fitness*)) OR exercising OR sitting OR sedentar* OR sport* OR ((Strength OR resistance*) ADJ3 training) OR (physical* ADJ3 education*) OR sleep* OR Insomni* OR (health ADJ3 (promotion* OR public*) ADJ6 (intervention* OR campaign*))).ab,ti,kw. OR (stress OR ((public* OR promotion*) ADJ3 health*)).ti.) AND (* Ethics/ OR exp * Ethics, Medical/ OR exp * Bioethics/ OR exp * Ethical Review/ OR exp * Ethical Analysis/ OR ((ethic*) ADJ3 (framework* OR frame-work*)).ab,ti,kw. OR ethic*.ti.) AND (((ethic*) ADJ3 (framework* OR frame-work*)).ab,ti,kw. OR (framework* OR frame-work*).ti.) AND (english.la. OR dutch.la.)

Embase

('lifestyle modification'/mj/de OR 'lifestyle intervention'/mj/de OR 'healthy lifestyle'/mj/de OR lifestyle/mj/de OR (lifestyle* OR life-style*):ti) AND (ethics/mj/de OR 'medical ethics'/mj/de OR 'virtue ethics'/mj/de OR 'bioethics'/mj/de OR 'ethical decision making'/mj/de OR ethic*:ti) NOT ([Conference Abstract]/lim OR [Conference Review]/lim) AND ([english]/lim OR [dutch]/lim)

('lifestyle modification'/de OR 'lifestyle intervention'/de OR 'healthy lifestyle'/de OR 'tobacco use'/exp OR 'smoking cessation'/exp OR 'health behavior'/de OR 'cigarette'/exp OR diet/exp OR 'food intake'/de OR drinking/de OR eating/de OR 'eating habit'/de OR malnutrition/de OR macronutrient/de OR 'trace element'/de OR 'macronutrient intake'/de OR 'micronutrient intake'/de OR 'dietary intake'/exp OR obesity/exp OR carbohydrate/exp OR nutrition/de OR exercise/exp OR 'physical activity'/exp OR sitting/de OR sport/de OR 'physical education'/de OR sleep/exp OR 'sleep disorder'/exp OR 'sleep parameters'/exp OR 'public health'/mj OR 'health promotion'/mj OR 'public health campaign'/exp OR (lifestyle* OR life-style* OR smoking OR tobacco* OR cigarette* OR diet* OR eating OR drinking OR (food* NEAR/3 intake*) OR malnutrit* OR macronutrient* OR trace-element* OR micronutrient* OR Meal-patter* OR ((calor* OR sugar* OR fat OR Protein* OR alcohol*) NEAR/3 (intake* OR consumption*)) OR obes* OR overweight* OR body-weight OR body-mass OR bmi OR carbohydrate* OR Vegetarian* OR vegan* OR Low-carb* OR nutrition* OR Binge-drink* OR (Alcohol* NEAR/3

abuse) OR alcoholism* OR (physical* NEAR/3 (activ* OR inactiv* OR exercise OR fitness*)) OR exercising OR sitting OR sedentar* OR sport* OR ((Strength OR resistance*) NEAR/3 training) OR (physical* NEAR/3 education*) OR sleep* OR Insomni* OR (health NEAR/3 (promotion* OR public*) NEAR/6 (intervention* OR campaign*))):ab,ti,kw OR (stress OR ((public* OR promotion*) NEXT/3 health*)):ti) AND (ethics/mj/de OR 'medical ethics'/mj/de OR 'virtue ethics'/mj/de OR 'bioethics'/mj/de OR 'ethical decision making'/mj/de OR ((ethic*) NEAR/3 (framework* OR frame-work*)):Ab,ti,kw OR ethic*:ti) AND (((ethic*) NEAR/3 (framework* OR frame-work*)):Ab,ti,kw OR (framework* OR frame-work*):ti) NOT ([Conference Abstract]/lim OR [Conference Review]/lim) AND ([english]/lim OR [dutch]/lim)

Web of science

TI=(((lifestyle* OR life-style*)) AND (ethic*)) AND DT=(article) AND LA=(English OR dutch)

(TS=(lifestyle* OR life-style* OR smoking OR tobacco* OR cigarette* OR diet* OR eating OR drinking OR (food* NEAR/2 intake*) OR malnutrit* OR macronutrient* OR trace-element* OR micronutrient* OR Meal-patter* OR ((calor* OR sugar* OR fat OR Protein* OR alcohol*) NEAR/2 (intake* OR consumption*)) OR obes* OR overweight* OR body-weight OR body-mass OR bmi OR carbohydrate* OR Vegetarian* OR vegan* OR Low-carb* OR nutrition* OR Binge-drink* OR (Alcohol* NEAR/2 abuse) OR alcoholism* OR (physical* NEAR/2 (activ* OR inactiv* OR exercise OR fitness*)) OR exercising OR sitting OR sedentar* OR sport* OR ((Strength OR resistance*) NEAR/2 training) OR (physical* NEAR/2 education*) OR sleep* OR Insomni* OR (health NEAR/2 (promotion* OR public*) NEAR/5 (intervention* OR campaign*))) OR TI=(stress OR ((public* OR promotion*) NEAR/2 health*))) AND (TS=((ethic*) NEAR/2 (framework* OR frame-work*)) OR TI=ethic*) AND (TS=((ethic*) NEAR/2 (framework* OR frame-work*)) OR TI=(framework* OR frame-work*)) AND DT=(article) AND LA=(English OR dutch)
