# Supplementary material for: Creating a Basic Ethical Framework for Digital Lifestyle Interventions: A Narrative Review
Source: Mayo Clin Proc Digit Health. 2025 Oct 14;3(4):100295. doi: 10.1016/j.mcpdig.2025.100295 (PMC12648102; doi:10.1016/j.mcpdig.2025.100295)
Supplement: Supplemental Table 1 [file mmc4.pdf]

Supplemental Table 1

Included Articles and Justification

General Ethical Principles for Public Health Interventions

Author[s]	Journal	Year	Title	Reason for inclusion
E. Kass	American Journal of Public health	2001	An ethics framework for public health	Proposes a foundational ethical framework specifically tailored to public health practice.
G. Marckmann et al.	Frontiers in Public health	2015	Putting public health ethics into practice: a systematic framework	Provides a practical tool for applying ethical analysis in real-world public health settings.
A. Tannahill	Health Promotion International	2008	Beyond evidence – to ethics: a decision-making framework for health promotion, public health and health improvement	Introduces a decision-making model integrating ethical reasoning in public health.
N. Baum et al.	Journal of Law and Medical Ethics	2007	Looking Ahead: Addressing Ethical Challenges in Public Health Practice	Addresses future ethical issues in public health with guidance for practitioners.
J. Childress et al.	Medical Ethics	2002	Public Health Ethics: Mapping the Terrain	Defines key ethical principles and distinguishes public health ethics from clinical ethics.
F. Filitrault & M. Désy	Not applicable	2017	Framework of Values to Support Ethical Analysis of Public Health Actions	Presents a values-based framework developed by a national public health institute.
Public Health Leadership Society	Not applicable	2002	Principles of the Ethical Practice of Public Health	Lists professional ethical standards widely adopted in

				U.S. public health practice.
Nuffield Council on Bioethics	Not applicable	2007	Public Health: ethical issues	Provides a ladder of intervention and ethical framework used internationally.

Ethical Frameworks for Lifestyle Interventions

Author[s]	Journal	Year	Title	Reason for Inclusion
L. Breunis et al.	Nicotine and Tobacco Research	2020	Incentives for smoking cessation during pregnancy: An ethical framework	Proposes an ethical framework specific to incentive-based interventions during pregnancy.
T. Have et al.	European Journal of Public Health	2013	An ethical framework for the prevention of overweight and obesity: a tool for thinking through a programme's ethical aspects	Introduces a structured ethical tool for evaluating public health programs targeting obesity.

Ethical Considerations for Digital Tools in Health Promotion

Author[s]	Journal	Year	Title	Reason for Inclusion
C. Brall et al.	European Journal of Public Health	2019	Ethical aspects of digital health from a justice point of view	Focuses on equity and distributive justice in digital health access.
L. Segura Anaya et al.	Science and Engineering Ethics	2018	Ethical implications of user perception of wearable devices	Addresses ethical issues of digital tools from the user perception side, including trust and privacy.
P. Nickel et al.	Digital Society	2023	Justice and empowerment through digital health: Ethical challenges and opportunities	Discusses how digital health can either reinforce or reduce health inequalities.

A. Koh et al.	Health Promotion International	2021	Digital health promotion: promise and peril	Explores the potential ethical risks of digital tools in health promotion.
N. Maher et al.	International Journal o Medical Informatics	2019	Passive data collection and use in healthcare: A systematic review of ethical issues	Synthesizes key ethical concerns related to non-consensual (secondary) data capture and use by digital tools.
E. Vayena et al.	Swiss Medical Weekly	2018	Digital health: Meeting the ethical and policy challenges	Provides ethical analysis and policy guidance for digital health innovation.
